# From Bits of Data to Bits of Knowledge—An On-Board Classification Framework for Wearable Sensing Systems

**DOI:** 10.3390/s20061655

**Published:** 2020-03-16

**Authors:** Pawel Zalewski, Letizia Marchegiani, Atis Elsts, Robert Piechocki, Ian Craddock, Xenofon Fafoutis

**Affiliations:** 1 Department of Electrical & Electronic Engineering, University of Bristol, Bristol BS8 1UB, UK; pz14094.2014@my.bristol.ac.uk (P.Z.); r.j.piechocki@bristol.ac.uk (R.P.); ian.craddock@bristol.ac.uk (I.C.); 2 Department of Electronic Systems, Aalborg University, Aalborg Ø 9220, Denmark; lm@es.aau.dk; 3 Institute of Electronics and Computer Science (EDI), Dzerbenes 14, Riga LV-1006, Latvia; 4 Department of Applied Mathematics and Computer Science, Technical University of Denmark (DTU), Kgs. Lyngby 2800, Denmark; xefa@dtu.dk

**Keywords:** wearable systems, embedded machine learning, embedded classifiers, intelligent duty-cycling, health IoT

## Abstract

Wearable systems constitute a promising solution to the emerging challenges of healthcare provision, feeding machine learning frameworks with necessary data. In practice, however, raw data collection is expensive in terms of energy, and therefore imposes a significant maintenance burden to the user, which in turn results in poor user experience, as well as significant data loss due to improper battery maintenance. In this paper, we propose a framework for on-board activity classification targeting severely energy-constrained wearable systems. The proposed framework leverages embedded classifiers to activate power-hungry sensing elements only when they are useful, and to distil the raw data into knowledge that is eventually transmitted over the air. We implement the proposed framework on a prototype wearable system and demonstrate that it can decrease the energy requirements by one order of magnitude, yielding high classification accuracy that is reduced by approximately 5%, as compared to a cloud-based reference system.

## 1. Introduction

By 2050, more than a quarter of the world’s population will consist of the elderly [1]. As a result, healthcare systems will struggle to meet an ever-increasing demand worldwide. This challenge can be addressed by automating some of the health assessment tasks, which in turn would reduce the strain on the healthcare systems and allow them to use their resources more efficiently [2]. In this context, Health IoT (Internet of Things) technology, such as wearable sensors, provides the foundation for long-term behavioural monitoring, enabling data scientists to mine the data, and use classification algorithms to extract knowledge that could be later analysed by qualified medical experts [3].

Traditional approaches to residential monitoring and behavioural analytics are based on raw data collection from one or more sensing elements, followed by cloud-based post-collection analysis (e.g., Reference [3]). For example, many activity recognition frameworks are designed and evaluated on carefully collected and annotated public datasets [4]. However, in practice, data loss due to poor device maintenance is a possibility; if the patient forgets to charge the battery then there might be days or even weeks of no data output from the wearable device. For real-world examples, see Reference [5], which discusses experiences from deployments of more than 100 wearable devices that demonstrate that a large number of participants fail to comply with simple recharging instructions.

Abundant data collection is expensive in terms of energy as the on-board radio is heavily used. The situation is most severe when using wearable sensors that employ particularly energy-consuming sensing elements. In activity-focused wearable sensors, for instance, accelerometers and gyroscopes are typically used. A general observation is that, whilst the former can be ultra-low power, the energy the latter require to operate is comparable to processors and wireless radios.

We argue that, in these contexts, the accuracy and the energy requirements of the knowledge extraction process should be jointly considered. Optimising for short-term accuracy may easily result to short battery lifetime, thus to data loss due to poor device maintenance, and, in turn, to poor long-term performance. Instead, it may be preferable to accept a small reduction of the short-term accuracy to save energy, and sustain higher performance in the long run.

**Figure 1:**
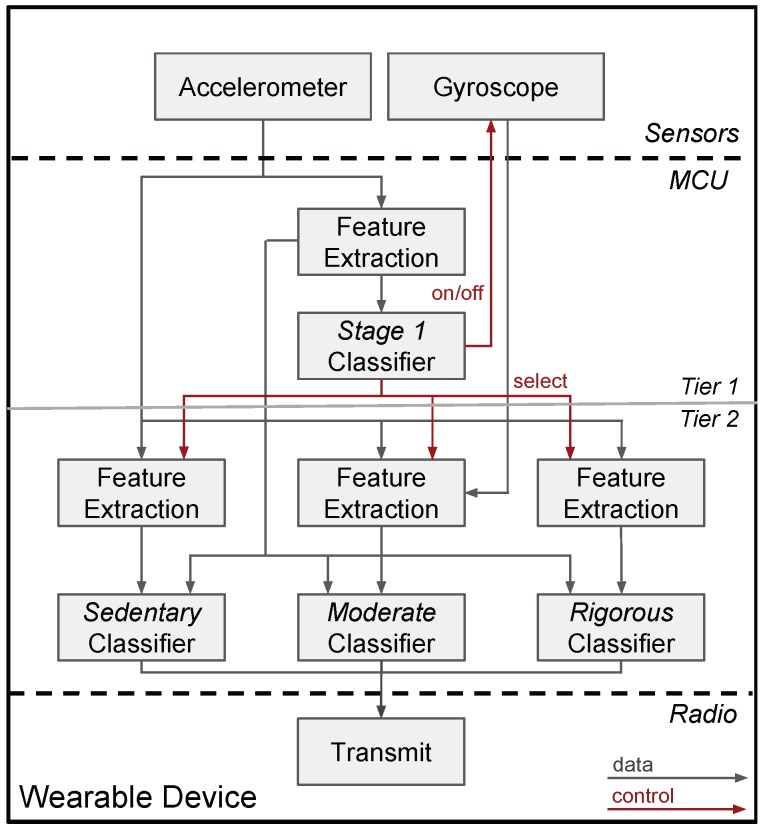
The proposed framework for on-board activity classification with wearable sensing systems. The framework is composed of an series of Random Forest classifiers that are structured hierarchically in two tiers. In the top tier (Tier 1), the Stage 1 classifier identifies the activity group (Sedentary, Moderate and Rigorous activity groups) and *selects* the corresponding Tier 2 classifier, which performs the activity classification. Only one of the Tier 2 classifiers and its corresponding feature extraction block is activated at a time depending on the output of the Stage 1 classifier. This approach allows us to keep the energy-hungry gyroscope powered-off, and activate it only when the user engages in activities whose classification can benefit from it. It is also noted that every feature extraction block extracts the features that are most informative for the respective classification task.

In this spirit, this paper proposes a framework for on-board classification of activities of daily life using wearable sensing devices, as summarised in Figure 1. The proposed framework decreases the energy requirements of a wearable sensing device in two ways. Firstly, on-board classification naturally decreases the amount of data transmitted over the air, and hence the radio duty cycle; effectively, the device communicates few bits of knowledge instead of streams of raw data. We demonstrate in this paper that the benefits from reducing the radio duty cycle vastly outweigh the cost of increasing the processor duty cycle. Secondly, the proposed framework is organised in an hierarchy of classifiers. This allows us to intelligently duty-cycle any power-hungry sensors, such as a gyroscope, in a context-aware manner. Initially, a first classifier identifies the activity group, using solely data from the ultra-low power accelerometer, which operates at 100% duty cycle. Based on this initial decision, a second classifier is selected for the activity recognition task, and the gyroscope is enabled only when the user is engaged in activities whose classification could benefit from it.

The proposed on-board classification framework is fully implemented on a wearable prototype. In turn, we profile its energy requirements and we benchmark it against the conventional cloud-based approach. The results demonstrate an improvement in the device lifetime by one order of magnitude. In addition, the proposed framework sustains high classification accuracy, yet the energy improvements come at the cost of approximately 5% accuracy, as compared to the reference cloud-based system.

The remainder of this paper is structured as follows. Section 2 briefly summarises the related work. Section 3 focuses on the design of the proposed framework. Section 4 offers details on the implementation and evaluation of the proposed framework. Finally, Section 5 concludes this paper.

## 2. Related Work

The proposed framework combines concepts, such as intelligent sensor duty cycling, hierarchical classification, feature engineering, and embedded machine learning, among others. These concepts have previously appeared in the literature in different contexts. Indeed, the framework’s novelty lies not on its parts, but in their unique combination into a system that trades a little short-term accuracy for a massive reduction of the energy consumption. We are particularly interested in quantifying this trade-off and we argue that this trade is, in several use cases, beneficial for severely resource-constrained devices, as it has the potential to lead to sustainable accuracy in the long run, through a massive reduction of the maintenance overhead, and thus the data loss due to poor device maintenance.

Embedded machine learning is casually adopted in smartphones ([6,7]), CCTV cameras (e.g., Reference [8]) and robots (e.g., Reference [9]). The concept of hierarchical classification itself has been proposed for sound classification in the context of smartphones [10] and smart vehicles [11]. In turn, intelligent duty cycling of high-power sensing elements, such as gyroscopes, GPS receivers and cameras, has been proposed as a means to extend the battery lifetime of smartphones [12,13] and mobile robots [14]. Different to these works, we target severely resource-constrained devices [15] (20kB RAM, 128kB Flash, and energy budgets of approximately 100 mAh).

Severely constrained devices are characterised by the *cost-accuracy conflict*. The trade-off between accuracy and cost was analysed in Reference [16], whereby several models were compared and the cost of model implementations was assessed in clock ticks. More recently, Reference [17] studied this trade-off from the perspective of feature engineering, providing measurements on the energy cost and value of various accelerometer features for activity recognition. Our system builds on these findings. In the same spirit, Reference [18] investigates the cost-accuracy trade-off by dynamically adapting the sampling frequency.

In the context of resource-constrained environments, embedded machine learning has been adopted in a variety of applications. Suresh et al. [19] employ a simplified kNN classifier for on-board classification in animal farming. Ravi et al. [20] employ kNN and a Bayesian classifier for detection of mosquito populations. Embedded machine learning has been used for the improvement of protocols, such as classification of wireless interference [21] and self-adapting MAC protocols [22]. On-board classification was also used to extend the lifetime of a wearable sensor that tracks physical activity levels [23]. Different to these works, our focus in not on the application itself; but rather on investigating and quantifying the trade-off between short-term accuracy and device lifetime.

Pedram et al. [24,25] employ embedded cascaded binary SVN classifiers for activity recognition, demonstrating that an hierarchical architecture is more efficient than multi-class classification. Our work employs a similar architecture of hierarchical classifiers; yet, different to References [24,25] we use the hierarchical architecture to intelligently duty cycle the gyroscope to save energy. These works do not duty cycle the gyroscope. In addition, we are interested in reducing the overall energy consumption of the system using a combination of techniques (i.e., feature engineering, intelligent sensor duty cycling, reduction of radio duty cycle, model reduction, and hierarchical classification) and investigating the cost-accuracy trade-off. Moreover, these works do not take into account the energy consumption of the radio and the communication-computation trade-off.

Finally, a recent trend in embedded machine learning for IoT devices includes the use of hardware accelerators for neural networks. Examples can be found in academic research [26,27,28] and off-the-shelf industrial solutions [29]. Although leveraging such hardware is important whenever available, our work targets wearable systems with no special hardware capabilities; hence, the proposed framework operates on general-purpose microcontrollers and is backwards-compatible to legacy IoT systems.

## 3. Framework Design

This section describes the design of the on-board classification framework. Initially, we provide details on data collection (Section 3.1). In turn, we discuss the classifier (Section 3.2) and feature extraction (Section 3.3). Next, Section 3.4 provides reference models assuming a cloud-based post-collection approach (off-board classification). Lastly, Section 3.5 provides reduced models suitable for on-board implementation (on-board classification).

### 3.1. Input Dataset

The models are trained and tested on a dataset collected with a custom prototype wearable sensor. The proposed on-board classification framework is also implemented on the same device. The system is based on the CC2640R2 (ARM Cortex-M3) and incorporates two inertial sensors: the triaxial MC3672 accelerometer [30] and the ICM20948 Inertial Monitoring Unit (IMU) [31], which is used as triaxial gyroscope. The two sensors have their axes co-aligned. The MC3672 accelerometer is connected with the MCU over Serial Peripheral Interface (SPI) at 4 MHz, and it is configured to work in the ±8 g amplitude range at 12 bits per sample. The sampling frequency is 14 Hz. The ICM20948 gyroscope is interfaced with the MCU via an SPI bus at 7 MHz. It is configured to operate at 17 Hz with 16 bits of resolution. It is noted that the sensors cannot be configured at the same sampling frequency due to hardware limitations. Instead, the samples are collected at 17 Hz and padding with data repetition is used when necessary. The chosen sampling rate is sufficient for human activity recognition as shown in Reference [32]. It is also highlighted that the gyroscope requires three orders of magnitude more power than the accelerometer according to their datasheets. This is a typical observation in activity-based wearable sensors [32].

The collected data include samples of 3 sedentary (i.e., sitting, lying, standing), 3 moderate (i.e., walking, turning leftwards, turning rightwards), and 3 rigorous (i.e., jumping, running, exercising) activities. These are representative activities of daily life that are commonly selected in the literature (e.g., References [6,7,33,34]). Aiming to use this dataset to evaluate the proposed on-board classification framework, we have included activities that have a strong rotational component. It is noted that an accelerometer-only framework would be more suitable for activities that do not exhibit rotational components.

Data was collected from seven volunteers, aged between 23 and 36 years, 3 females and 4 males. The wearable sensor was attached to their wrist; the participants were free to choose which arm to use during the experimentation process. The participants were asked to perform one activity at a time in a loop for 2.5 min, which results to 17.5 min of data per activity for each sensor. To maximise the variance in the data, no particular instructions were given to the subjects as to where or how to execute the activities.

We highlight that the problem of recognition of these nine activities represents a *use case* that we adopt to evaluate the proposed framework. Indeed, the framework can be easily adapted to different activities, different classification tasks, as well as different sensing modalities.

It is also noted that recent literature in activity recognition is looking into more realistic in-the-wild data collection to improve the generalisation power of the classification process [3]. While we appreciate their aspirations, we opted for a dataset of loosely scripted activities, considering it sufficient for the objectives of this work. Indeed, our objective is not to provide a solution that outperforms the activity recognition state-of-the-art in absolute terms, but rather to quantify and demonstrate the benefits of our on-board classification framework, as opposed to traditional cloud-based approaches.

### 3.2. Classification

The classification process is conducted in two steps, as introduced in Figure 1. The first step, namely Stage 1, is the classification according to the energy band of the physical activity, namely Sedentary, Moderate, and Rigorous. The second step depends on the result of the first step, and aims to classify the activities within the particular energy band. The primary motivation behind this hierarchical approach is to activate the expensive gyroscope only when needed. Moreover, doing so allowed us to work on four simpler models instead of one complex model, but this entails that the Stage 1 classifier must be very accurate as any errors will be propagated further to the classifiers that are at the second level.

The proposed framework is based on Random Forest: a probabilistic classifier that is composed of an ensemble of decision tree classifiers [35]. For the purposes of this work, we also considered the k-Nearest Neighbours (kNN), Support Vector Machine (SVM), and Deep Neural Network (DNN) classifiers. All four classifiers yield comparable results in terms of accuracy (see Reference [36] for detailed results). Ultimately, we selected Random Forest. The main reason is feasibility for efficient on-board implementation for general-purpose micro-controllers, similar to Reference [21]. It is noted that DNN models can be efficiently executed in low-power platforms, but this requires specialised hardware that may not be available on all wearable platforms.

### 3.3. Feature Extraction

As the ultimate goal of this work is to implement the process on a resource-constrained embedded system, the full feature space is based on features that are cheap in terms of computational requirements. In particular, good balance between accuracy and extraction cost is provided by basic time-domain features [17,37], which include: (i) maximum; (ii) median; (iii) minimum; (iv) mean; (v) and variance of the data sequence along the *x*, *y* and *z* axes. Such features, indeed, provide a characterisation of the central tendency of the data distribution (e.g., mean, median), as well as of its dispersion (e.g., maximum, minimum). These time domain features are calculated over a window *w* for each of the three axes, resulting to 15 features per sensor. In addition to the above, we also employ the Integral of the Modulus of Acceleration (IMA), calculated as in Reference [23].

We use a window size of 1.6 s. This is selected to match the memory buffer of the accelerometer for energy-efficient design. Nevertheless, this window size is in accordance with References [32,38], which advocate for a window of 1–2 s. Moreover, the features are computed over a 50% overlapping window, capturing the temporal nature of the activities [17]. The selected window size results to a total of 9000 samples, 1000 samples in each class.

### 3.4. Full Models for Off-Board Classification

In this section, we provide reference models that are trained for cloud-based off-board classification. This step provides a point of reference of the maximum possible accuracy.

#### 3.4.1. Accelerometer Only

We first investigate the performance of a Random Forest classifier of 100 trees using the data from the accelerometer and all 16 features. The input feature vectors (i.e., statistics over the specified windows) are randomly divided into training and test sets (50%–50%), keeping a balanced class representation. The process is repeated 1000 times on different random training and test sets. Table 1 shows the accuracy of each stage. The results demonstrate high accuracy in all tasks apart from the Moderate stage.

#### 3.4.2. Engaging the Gyroscope

Next, the classifiers are retrained using features from both the accelerometer (16 features) and the gyroscope (15 features). The results (Table 2) show that enabling the gyroscope yields marginal improvements in all cases but the Moderate stage, in which the gyroscope improves the mean accuracy by 15.3%.

### 3.5. *Reduced* Models for On-Board Classification

An on-board implementation of the full models would be impractical due to memory and energy constraints. In this section, we provide classification models that are suitable for implementation on wearable devices. This is achieved by reducing the number of features, the number of trees, and the maximum number of splits (i.e., the maximum tree depth). Our goal is to decrease the requirements of the classifiers in terms of resources without introducing significant performance loss in terms classification accuracy. For the remainder of this paper, we will refer to them as *reduced* models.

#### 3.5.1. Reducing the Number of Features

**Figure 2:**
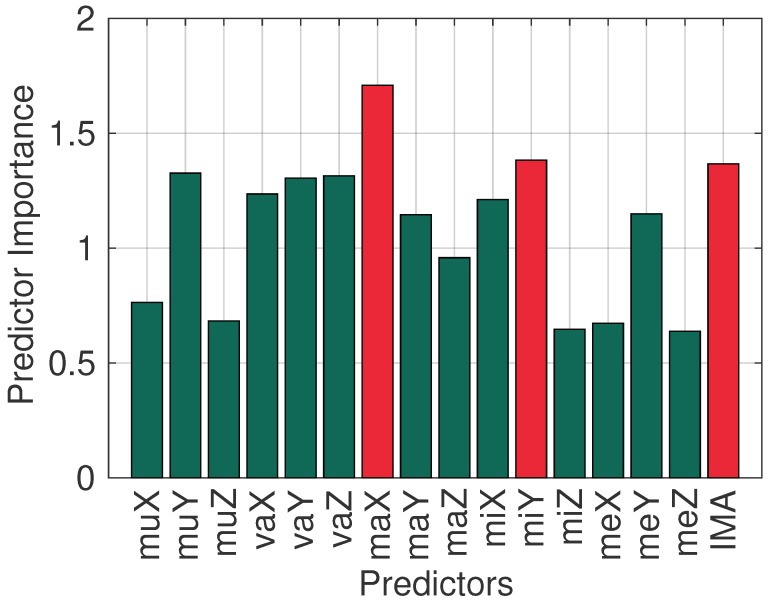
Feature importance in Stage 1, for features mean (mu), variance (va), maximum (ma), minimum (mi), and median (me). X,Y,Z correspond to the respective axis.

Focusing on the accelerometer-only case, we investigate the impact of reducing the number of features, with the goal to reduce the energy cost of feature extraction. The Random Forest classifier provides a means to rank the features, also known as predictors, in terms of their importance. For example, Figure 2 plots the predictor importance for Stage 1, and demonstrates that most information is contained in three features: the maximum value of the X axis, the IMA, and the minimum of the Y axis.

We reduce the number of features of each classifier, based on the information gain ranking table provided by the Random Forest. In particular, to allow for an energy-efficient on-board implementation, we limit the maximum number of features to N=8. Otherwise, we use the first n<N features that provide an accuracy that is less than 2% of the accuracy achieved with the full feature set. It is highlighted that any feature that is extracted during Stage 1 can be used in the second stage without any additional energy costs.

Table 3 provides the classification accuracy assuming the reduced feature space. The results show that the reduced features space yields to an insignificant gain in Stage 1, minor losses in the Sedentary and Moderate cases, and a more considerable loss of 5.1% in the Rigorous case.

#### 3.5.2. Reducing the Number of Trees

**Figure 3:**
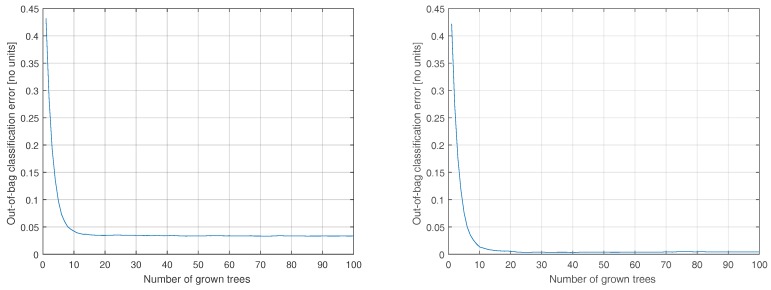
The effect of decreasing the number of trees on the accuracy of the Stage 1 classifier (**left**) and Sedentary classifier (**right**). 10 trees is a reasonable compromise between accuracy and feasibility for on-board implementation.

**Figure 4:**
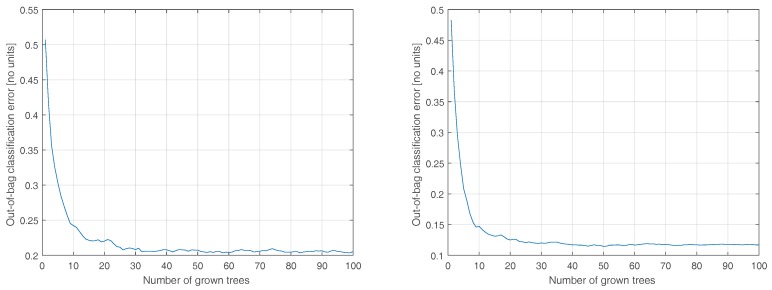
The effect of decreasing the number of trees on the accuracy of the Moderate classifier (**left**) and Rigorous classifier (**right**). 10 trees is a reasonable compromise between accuracy and feasibility for on-board implementation.

Next, we investigate the effect of number of trees on the accuracy. To this end, we repeat the process considering 1 to 100 trees. The results for the Stage 1 classifier are shown in Figure 3 (left). The results suggest diminishing returns with marginal benefits in growing more than 15 trees for the collected data and, indeed, 10 trees is satisfactory relative to the performance of the unconstrained model. The same pattern is observed in the case of the Tier 2 classifiers, shown in Figure 3 (right) and Figure 4, yet the Moderate classifier can benefit from up to 30 trees. Interested in an energy-conscious implementation, for the remainder of this paper, we fix the number of trees at 10 for all four on-board classifiers.

#### 3.5.3. Reducing the Number of Splits

The next step is to reduce the number of splits (nodes) in each tree. As shown in Table 4, with 50 iterations, the classifiers reach high accuracy at approximately 20 splits with marginal improvements beyond this. Yet, a large number of splits it is not practical for an embedded implementation, due to the increased number of nodes. Therefore, in the reduced models for on-board implementation, the number of splits was constrained to maximum 5. In addition, each classifier is trained with the reduced feature space and with the number of trees limited to 10. As we also discuss in Section 4, the full system implementation uses 98% of the available memory, highlighting that the memory imposes a constraint on the number and size of the trees. The results of this process are summarised in Table 5. The last column shows the performance degradation compared to 100 trees, also trained on the reduced feature space. It can be seen that the performance is reduced most severely in the Moderate and Rigorous stages.

#### 3.5.4. Engaging the Gyroscope

Next, we investigate the influence of the gyroscope on the accuracy of the on-board classification task. The following classifiers were trained using the reduced feature set from the acceleration sensor and the full feature set from the gyroscope. The classification results are presented in Table 6.

The gains of employing both peripherals are quite limited apart from the case of distinguishing turning leftwards from turning rightwards. Indeed, there are no substantial gains for Stage 1, only 3.1% for Sedentary, and 4.5% for Rigorous; yet a great gain of 29.2% for the Moderate case. Considering that a gyroscope is much more energy-consuming than ultra-low power accelerometers (by up to three orders of magnitude), these results suggest that we can activate the gyroscope only when Stage 1 identifies that the user engages in an activity that would significantly benefit it (i.e., Moderate in our use case).

To further reduce the energy consumption, we next reduce the feature set of the gyroscope. The results indicate that most of the information is contained in the mean of the *X* axis, and in fact adding this single feature into the reduce acceleration feature set yields an accuracy of 93.5% in the Moderate case. It is worth mentioning that this reduced model of the Moderate classifier is still performing better than the full model derived solely from accelerometer data by 22.1%.

#### 3.5.5. Summary

This section has described how a complex and accurate model can be downsized to a functional bare-bones version by feature engineering, tuning of the hyper-parameters and trading off some of the algorithm’s accuracy for plainness. To provide further insight on the performance of the reduced classifier intended for on-board implementation, we provide confusion matrices for the four stages in Table 7. The matrices contrast the predictions (output) against the ground truth (target). The diagonals denote the percentage of correct predictions in each case. It is noted that, based on the presented results, the gyroscope is used only when Stage 1 predicts that the user engages in a Moderate activity. As a result, the confusion matrices of the Stage 1, Sedentary, and Rigorous classifiers correspond to the case of using the acceleration sensor only. The matrix of the Moderate classifier, instead, corresponds to the case of using both sensors.

## 4. Implementation and Evaluation

### 4.1. Implementation

We next implement the proposed on-board classification framework for the CC2640R2 (ARM Cortex M3 processor), incorporating the four reduced classifiers, as shown in Figure 1.

The implementation fully exploits the FIFO (First In First Out) memory that is embedded with the MC3672 accelerometer. This allows us to store 32 samples at 12-bit resolution. This approach reduces the wake-up events of the processor, which are costly for the power budget of the system as the chip is woken up from 1.1 μA standby mode into 3 mA active mode. In addition, the data collection was implemented with a 16-sample overlap between adjacent windows. Each 32-sample window of raw data is, in turn, passed for feature extraction, and then to the Stage 1 classifier. The data is, in turn, passed to the respective second stage classifier, upon any additional feature extraction if necessary. The gyroscope is activated only in the Moderate case. This implies that the device is enabled *after* the Stage 1 classifier has detected that the system is in the Moderate state. Hence, the samples it provides are not available until the next sampling window. As a result, we implemented two versions of the Moderate model: one that works only with the accelerometer data (accuracy of 66.7%), and one that works with both sensors (accuracy of 93.3%). The effect of this solution is incorporated on the final results. Once activated, the gyroscope is operating in the exact same manner as the accelerometer. Finally, the output is transmitted over BLE advertisements.

A 32-sample window of raw data is 192 bytes. At the end of the processing chain, this value is reduced to 4 bytes: 1 byte to encode the state of the energy class (output of Stage 1) and the most likely class within each band (output of respective Stage 2); 3 bytes contain information regarding the posterior probability that the sample belongs to each activity class. The framework is compressing the data with a ratio of 1:48.

The full system implementation uses 98% of the available flash memory (124.46 kB). This highlights the existence of severe memory constraints, which pose a natural limit on reduction parameters, such as the number of trees.

### 4.2. Energy Consumption

For evaluating the energy consumption of the proposed on-board classification framework we adopt the methodology presented in Reference [39]. In particular, the energy consumption is approximated by combining energy measurements of isolated events with timing measurements on the frequency and duration of these events. The timings are measured using the on-board 48 MHz clock. We highlight that uncontrolled energy measurements are impractical, because the users would need to wear the device and engage in potentially rigorous activities, whilst the device is wired to a power analyser.

#### 4.2.1. Feature Extraction

Table 8 represents the costs associated with transferring data within the wearable system, which includes reading 192 bytes form the FIFO, reading a single sample, converting the 192 bytes data into 32 triaxial samples, and all operations required for copying the data to radio transmission buffer. The device drains approximately 3.5 mA when in active mode and accounting all the peripherals, which translates to 12.95 mW of power consumption. Additionally, when the gyroscope is enabled the current usage is 4.57 mA and the power consumption is increased to 16.9 mW. The read cost from the gyroscope is lower than the accelerometer, due to the SPI bus, which runs at 7 MHz and 4 MHz respectively.

Table 9 summarises the computation costs for feature extraction. The most expensive feature is the IMA, which operates on data from all three axes.

#### 4.2.2. Classification

The Random Forest models were implemented on the CC2640R2, each consisting of 10 trees and 5 splits per tree (250 if statements in total). The classifiers were trained using Gini impurity as splitting criterion and sampling is carried out with replacement. The minimum split sample size is 10 and the minimum leaf size of the four classifiers is 71, 143, 47 and 74 respectively. Due to the 50% window overlap, the classification process is executed at twice the frequency of a FIFO update. Thus, the hierarchical classifiers are executed every 1.15 s. Table 10 characterises the computation cost for executing each classifier.

#### 4.2.3. Radio

The BLE radio is using 6.1 mA when transmitting at 0 dBm, which entails 22.57 μW of power usage for the system. The power profile of transmitting an advertisement, measured on the high side of the power supply, indicates that the energy cost of a BLE advertisement is 73.16 μJ.

### 4.3. Latency

One of the advantages of edge computing is low latency via the elimination of the communication delays. In practice, the end-to-end latency of an embedded application depends on three components: the sensing delay, the communication delay and the processing delay. In the proposed on-board classification framework, the collection of one window of 32 samples in the FIFO requires approximately 2.3 s. Reading the FIFO requires less than 1 ms (see Table 8) and, thus, is considered negligible. The communication delay is zero as the classification is performed locally. Finally, the processing delay for feature extraction and classification is less than 2 ms (see Table 9 and Table 10). As a result, the end-to-end delay from the sensing the first sample of the window until classification is roughly 2.3 s. For comparison, a reference off-board architecture would transfer the data, one by one, to a central server for classification. In this case, the samples are collected one by one at 17 Hz, thus the process gets completed in approximately 1.9 s. The communication delay depends on the network topology (e.g., multi-hop vs single-hop) and the delay introduced by the employed wireless technologies. As an indicative example, let us assume the SPHERE smart home architecture [40], which is our application of interest. In this architecture, each data packet is first sent to a receiver node over BLE (roughly 2 ms). In turn, the packet is queued and transmitted over a TSCH (Time-Slotted Channel Hopping) mesh network of one or two hops. Assuming the packet is transmitted on the next slot frame, this step takes up to 1 s, that is, the duration of the slot frame of 100 slots (10 ms per slot), assuming no communication errors that would require re-transmissions in the next frame. The processing delay can be considered negligible as the processing power of a typical IoT gateway is much higher than a wearable sensor. In total, the end-to-end delay is approximately 2.9 s or more in case of re-transmission delays.

### 4.4. Device Lifetime

Research has shown that estimating the battery lifetime of a device from its energy consumption profile is not trivial, given the non-linear properties of the batteries [5,41]. In this section, we provide indicative battery lifetime estimations that are based on the methodology presented in Reference [39].

As a benchmark, we compare the implemented classifiers against the conventional way of raw data collection (off-board classification). In particular, we consider two cases: accelerometer only, and accelerometer and gyroscope. We assume a $E_{BAT}=1332$ J energy budget. This roughly corresponds to a wearable-sized Lithium-Polymer (Li-Po) battery.

The estimated device lifetime (*T*) can be approximated by:

(1) $$T=\frac{E_{BAT}}{P_{I}+P_{A}+P_{G}+P_{MCU}}\;,$$

where $P_{I}$ is the system’s idle power consumption, $P_{A}$ and $P_{G}$ is the average power consumed by the accelerometer and the gyroscope, and $P_{MCU}$ is the average power consumed by the CC2640R2. The power associated with the gyroscope is zero, $P_{G}=0$, when the gyroscope is powered off.

In the raw data case, the latter is given by:

(2) $$P_{MCU}=\left(E_{SPI_{A}}+E_{SPI_{G}}+E_{DATA}+E_{RF}\right)\times f_{IRQ}\;,$$

where $E_{SPI_{A}}$ and $E_{SPI_{G}}$ are the energies consumed for transferring a *single sample* via the respective SPI bus, $E_{DATA}$ is the energy associated with copying the data to the radio buffer, $E_{RF}$ is the energy used by the BLE radio, and finally $f_{IRQ}$ is the frequency of collecting the samples and sending the advertisement over the air. The rate is $f_{IRQ}=17$ Hz, i.e., the sampling rate of the gyroscope.

For our framework, $P_{MCU}$ takes the following form:

(3) $$P_{MCU}=\left(E_{T}+\sum_{i=0}^{n}E_{F_{i}}+E_{S_{1}}+E_{S_{2}}\right)\times f_{IRQ}\;,$$

(4) $$\mathrm{where}\;E_{T}=\frac{E_{SPI_{A}}+E_{SPI_{G}}}{2}+E_{BLE}+E_{DATA}\;.$$

Different from above, $E_{SPI_{A}}$ and $E_{SPI_{G}}$ are the energies consumed for transferring a *full FIFO* via the respective SPI bus, and $f_{IRQ}$ is the classification frequency, $f_{IRQ}=0.87$ Hz. The SPI terms are halved as they occur at half of the classification frequency $f_{IRQ}$. The summation over $E_{F_{i}}$ terms is representing the energy consumed for extracting the *n* features associated with each random forest algorithm. Finally, the $E_{S_{1}}$ corresponds to the energy consumed for executing the Stage 1 classifier and $E_{S_{2}}$ corresponds to the energy consumed for executing the respective Stage 2 classifier.

The overall energy consumption of the system depends on how often each Stage 2 classifier is engaged. We thus attempt to estimate it proportionally to how often an average person spends their day in each of the energy bands. To that end, we employ a dataset from the Avon Longitudinal Study of Parents and Children (ALSPAC) that is collected from approximately 50 individuals who each wore an acceleration-based wearable sensor for 10 days [42]. The dataset was assessed using the classification method described in Reference [23] and the results have shown that an average person spends 80.8% of the day engaging in Sedentary activities, 18.8% of the day engaging in the Moderate activities, and 0.4% of the day engaging in Rigorous activities. Using these statistics as weights (*w*), $E_{S_{2}}$ can estimated as the weighted sum of the Stage 2 classifiers:

(5) $$E_{S_{2}}=\sum_{i=0}^{3}w_{i}E_{S_{2}}^{\left(i\right)}.$$

The energy consumption of the gyroscope and feature extraction is estimated similarly.

### 4.5. Results and Discussion

The final results are summarised in Table 11, where the proposed framework is labelled as EML (Embedded Machine Learning), and the benchmark is labelled as RAW (Raw data collection). For completeness, two on-board classification cases are considered: accelerometer only, and accelerometer and gyroscope. The results show that, in a practical environment, our framework increases the lifetime by one order of magnitude compared to the benchmark (RAW): the accelerometer-based system has increased its lifetime from 12 to 111 days and the configuration that duty-cycles the gyroscope increases the device lifetime from 3 to 17 days. Likewise, the power usage was reduced from 1.2 mW to 136 μW and from 5.4 mW to 884 μW respectively.

**Figure 5:**
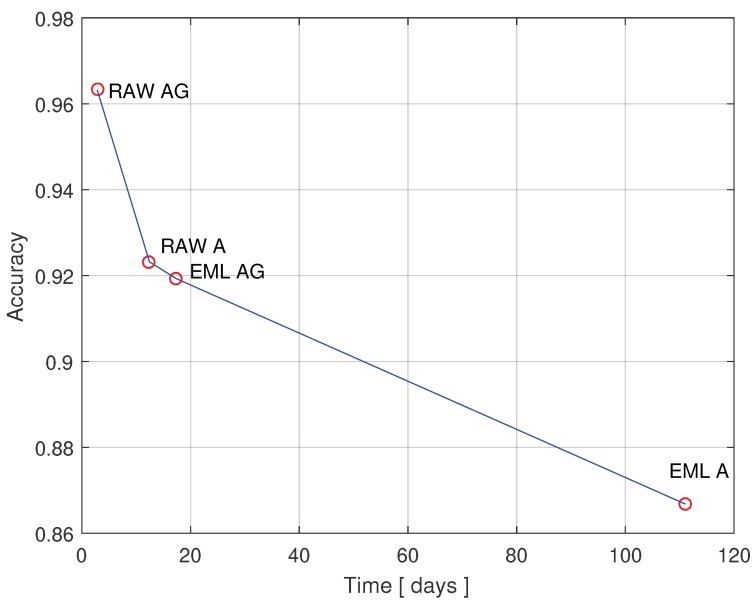
The trade-off between the model accuracy and lifetime. Legend: Raw Data—Accelerometer Only (RAW A); Raw Data—Accelerometer and Gyroscope (RAW AG); On-Board Classification—Accelerometer Only (EML A); On-Board Classification—Accelerometer and Gyroscope (EML AG).

The results obtained in this work confirm the trade-off between the short-term accuracy and the energy usage – the greatest accuracy is obtained when using the raw data, full sized models and all the features, whilst the most energy is saved when the models are reduced and only the most important features are exploited. In addition, the results quantify the trade-off, demonstrating that it is asymmetric. Table 11 also compares the accuracy of the full and reduced models. The overall weighted accuracy per model (*A*) reflects the fact that Stage 1 is always engaged first and that effectively the second stage is conditioned on the first stage, which can be represented using the equation:

(6) $$A=\alpha_{s_{1}}\sum_{i=1}^{3}w_{i}\alpha_{s_{2}}^{\left(i\right)}.$$

where $\alpha_{s_{1}}$ is the accuracy of the Stage 1 classifier; $\alpha_{s_{2}}^{\left(i\right)}$ is the accuracy of the *i*-th second stage classifier, namely Sedentary, Moderate, and Rigorous respectively; and $w_{i}$ is a ALSPAC weight that reflects the time each *i*-th second-stage classifier is engaged. The results demonstrate that the proposed framework improves the lifetime by an order of magnitude, sacrificing approximately 5% in classification accuracy. Finally, the data is plotted in Figure 5, which further illustrates the trade-off.

## 5. Conclusions

In Health IoT applications, the requirement to recharge wearable devices frequently is not only cumbersome, but may be downright unethical when monitoring ill or elderly people. Therefore it is vital to make the wearables as energy-efficient as possible to deliver the best user experience, to increase patient acceptance, and to avoid losing experimental data. We argue that obtaining lossless raw data from constrained devices in the wild is very expensive and, sometimes, impractical. Data loss due to poor device maintenance is inevitable, and therefore it is worth considering trading some short-term accuracy for a massive reduction of the maintenance overhead, aiming at sustaining high accuracy over time.

With this in mind, we propose an on-board classification framework for energy-efficient activity recognition using wearable sensors. The proposed framework increases the device lifetime by reducing the duty-cycle of the radio and the gyroscope. On one hand, the presented framework extracts knowledge from the raw data on the board, thus the information that needs to be transmitted over the air is significantly reduced. In parallel, our proposed solution is organised as a tiered ensemble of on-board classifiers, allowing the wearable to dynamically duty-cycle the energy-consuming gyroscope and use it only when it can provide significant contributions to the activity classification problem.

The proposed framework is fully implemented for a prototype wearable device that employs the ARM Cortex M3 processor, and compared against the conventional cloud-based classification approach. The comparison indicates that our solution has the potential to increase the lifetime of the wearable device by one order of magnitude, at only the cost of approximately 5% classification accuracy.

## Figures and Tables

**Table 1:** Off-Board Classification (accelerometer only).

Classifier	Accuracy, μ	*σ*
Stage 1	96.5%	0.2%
Sedentary	99.0%	0.1%
Moderate	81.4%	1%
Rigorous	93.2%	0.6%

**Table 2:** Off-Board Classification. The classification accuracy when using both the accelerometer and the gyroscope.

Classifier	Accuracy, μ	*σ*	Difference, Δμ
Stage 1	97.5%	0.2%	+1%
Sedentary	99.3%	0.3%	+0.3%
Moderate	96.7%	0.5%	+15.3%
Rigorous	94.9%	0.5%	+1.7%

**Table 3:** On-Board Classification (effect of feature reduction).

Classifier	Accuracy μ	*σ*	Difference, Δμ
Stage 1	96.6%	0.2%	+0.1%
Sedentary	98.7%	0.2%	−0.3%
Moderate	80%	1%	−1.4%
Rigorous	88.1%	0.7%	−5.1%

**Table 4:** On-Board Classification (effect of reduction of splits).

**Stage 1 Classifier**					
**Max. Splits**	**Accuracy, μ**	***σ***	**Nodes**	**Edges**	**Leaves**
5	95.6%	0.3%	11	10	6
10	96.3%	0.3%	21	20	11
20	96.6%	0.2%	41	40	21
30	96.8%	0.2%	61	60	31
40	96.9%	0.2%	81	80	41
50	96.9%	0.2%	101	100	51
**Sedentary Classifier**					
**Max. Splits**	**Accuracy, μ**	***σ***	**Nodes**	**Edges**	**Leaves**
5	97%	0.4%	11	10	6
10	99.2%	0.3%	21	20	11
20	99.6%	0.2%	41	40	21
30	99.7%	0.2%	57	56	29
40	99.7%	0.2%	59	58	30
50	99.7%	0.2%	59	58	30
**Moderate Classifier**					
**Max. Splits**	**Accuracy, μ**	***σ***	**Nodes**	**Edges**	**Leaves**
5	67.6%	1.2%	11	10	6
10	70.7%	1.4%	21	20	11
20	75.1%	1.3%	41	40	21
30	77%	1.1%	61	60	31
40	78.1%	1 %	81	80	41
50	78.7%	1.1%	101	100	51
**Rigorous Classifier**					
**Max. Splits**	**Accuracy, μ**	***σ***	**Nodes**	**Edges**	**Leaves**
5	81.5%	1 %	11	10	6
10	84.6%	0.7%	21	20	11
20	88.6%	0.8%	41	40	21
30	91.2%	0.9%	61	60	31
40	92.4%	0.7%	81	80	41
50	93.2%	0.6%	101	100	51

**Table 5:** On-Board Classification (effect of tree and split reduction).

Classifier	Accuracy μ	*σ*	Difference, Δμ
Stage 1	95.6%	0.5%	−1.0%
Sedentary	96.3%	0.5%	−2.4%
Moderate	66.7%	1.4%	−13.3%
Rigorous	80.0%	2.0%	−8.1%

**Table 6:** On-board Classification (engaging the gyroscope).

Classifier	Accuracy μ	*σ*	Difference, Δμ
Stage 1	97.4%	0.2%	+1.8%
Sedentary	99.2%	0.3%	+3.1%
Moderate	95.9%	0.7%	+29.2%
Rigorous	84.6%	2.0%	+4.6%

**Table 7:** Confusion matrices of the on-board classification framework.

**Stage 1** **F-Score 95.6%**		**Target**		
		**Sedentary**	**Moderate**	**Rigorous**
**Output**	Sedentary	**93.9%**	3.0%	0.1%
	Moderate	5.3%	**95.0%**	2.1%
	Rigorous	0.8%	2.0%	**97.9%**
**Sedentary** **F-Score: 96.3%**		**Target**		
		**Standing**	**Lying**	**Sitting**
**Output**	Standing	**100%**	0%	0%
	Lying	0%	**96.9%**	8.0%
	Sitting	0%	3.1%	**92.0%**
**Moderate** **F-Score: 92.8%**		**Target**		
		**Walking**	**Turning L**	**Turning R**
**Output**	Walking	**94.0%**	4.5%	4.9%
	Turning L	3.1%	**91.4%**	2.2%
	Turning R	2.8%	4.1%	**92.9%**
**Rigorous** **F-Score: 78.6%**		**Target**		
		**Exercise**	**Jumping**	**Running**
**Output**	Exercise	**78.9%**	6.3%	14.6%
	Jumping	14.2%	**81.0%**	9.4%
	Running	6.8%	12.7%	**76.0%**

**Table 8:** Peripheral operations cost.

Operation	Time [μs]	Energy [μJ]
MC3672 32-sample FIFO read	567.9	7.35
ICM20948 32-sample FIFO read	323.5	5.47
MC3672 single sample	38.4	0.50
ICM20948 single sample	22.0	0.37
Convert fifo to 16-bit int	36.9	0.48
Data preparation for the radio	927.6	12.01

**Table 9:** Feature computation cost over 32 samples.

Feature	Time [μs]	Energy [μJ]
IMA	628.3	8.14
Median	316.6	4.10
Variance	93.4	1.21
Mean	66.9	0.87
Maximum	12.6	0.16
Minimum	12.6	0.16

**Table 10:** Classifier computation cost.

Classifier	Time [μs]	Energy [μJ]
Stage 1	67.6	0.88
Sedentary	69.3	0.90
Moderate Acc	61.2	0.79
Moderate Acc + Gyr	108.3	1.41
Rigorous	71.0	0.92

**Table 11:** The Trade-off between Accuracy and Lifetime.

Configuration	Accuracy, μ	Power [μW]	Life [d]
RAW Acc	92.3%	1200	12
RAW Acc + Gyr	96.3%	5400	3
EML Acc	86.7%	136	111
EML Acc + Gyr	91.9%	884	17
